# Alfaxalone Anaesthesia Facilitates Electrophysiological Recordings of Nociceptive Withdrawal Reflexes in Dogs (*Canis familiaris*)

**DOI:** 10.1371/journal.pone.0158990

**Published:** 2016-07-19

**Authors:** James Hunt, Jo Murrell, David Knazovicky, John Harris, Sara Kelly, Toby G. Knowles, B. Duncan X. Lascelles

**Affiliations:** 1 School of Veterinary Sciences, University of Bristol, Bristol, United Kingdom; 2 Comparative Pain Research Laboratory, College of Veterinary Medicine, North Carolina State University, Raleigh, North Carolina, United States of America; 3 Division of Animal Sciences, School of Biosciences, University of Nottingham, Sutton Bonington Campus, Loughborough, United Kingdom; 4 Center for Pain Research and Innovation, UNC School of Dentistry, Chapel Hill, North Carolina, United States of America; 5 Comparative Medicine Institute, North Carolina State University, Raleigh, North Carolina, United States of America; Tokai University, JAPAN

## Abstract

Naturally occurring canine osteoarthritis represents a welfare issue for affected dogs (*Canis familiaris*), but is also considered very similar to human osteoarthritis and has therefore been proposed as a model of disease in humans. Central sensitisation is recognized in human osteoarthritis sufferers but identification in dogs is challenging. Electromyographic measurement of responses to nociceptive stimulation represents a potential means of investigating alterations in central nociceptive processing, and has been evaluated in conscious experimental dogs, but is likely to be aversive. Development of a suitable anaesthetic protocol in experimental dogs, which facilitated electrophysiological nociceptive withdrawal reflex assessment, may increase the acceptability of using the technique in owned dogs with naturally occurring osteoarthritis. Seven purpose bred male hound dogs underwent electromyographic recording sessions in each of three states: acepromazine sedation, alfaxalone sedation, and alfaxalone anaesthesia. Electromyographic responses to escalating mechanical and electrical, and repeated electrical, stimuli were recorded. Subsequently the integral of both early and late rectified responses was calculated. Natural logarithms of the integral values were analysed within and between the three states using multi level modeling. Alfaxalone increased nociceptive thresholds and decreased the magnitude of recorded responses, but characteristics of increasing responses with increasing stimulus magnitude were preserved. Behavioural signs of anxiety were noted in two out of seven dogs during recordings in the acepromazine sedated state. There were few significant differences in response magnitude or nociceptive threshold between the two alfaxalone states. Following acepromazine premedication, induction of anaesthesia with 1–2 mg kg^-1^ alfaxalone, followed by a continuous rate infusion in the range 0.075–0.1 mg kg^-1^ min^-1^ produced suitable conditions to enable assessment of spinal nociceptive processing in dogs, without subjecting them to potentially aversive experiences. This methodology may be appropriate for obtaining electrophysiological nociceptive withdrawal reflex data in client-owned dogs with naturally occurring osteoarthritis.

## Introduction

Osteoarthritis (OA) has been estimated to affect 20% of adult dogs (*Canis familiaris*), and the resulting pain presents a significant welfare challenge in the management of older animals [1]. Naturally occurring canine OA is most commonly secondary to joint dysplasia or abnormal loading forces and progresses over a time course. Canine OA shares developmental, pathological and clinical features with human OA [2]. In addition, client owned dogs are likely to be exposed to similar environmental conditions [3], and potentially lifestyle factors [4], as human OA sufferers. Spontaneously occurring canine OA has therefore been proposed as a model for further investigation of, and trial of therapeutics for the human condition, with the potential to impact both animal and human welfare [5]. If this model is to be optimized, all relevant outcome measures and measures of the pain state need to be developed and validated, including measures of central sensitisation.

The development of enhanced nociceptive transmission within the central nervous system (central sensitisation, CS) is a feature of OA in a subset of human sufferers [6], and may predict a poor response to present standard of care analgesics [7]. In humans CS can be characterised by the application of a variety of sensory modalities [7] and the presence of CS is indicated by secondary mechanical dynamic and punctate hyperalgesia. Central sensitisation has also been suspected in clinical canine OA patients [8]; however there is presently no standard method of assessing CS in dogs, therefore the fidelity of the spontaneous canine OA model to human OA cannot be assured. Furthermore, there is the potential for varying and unknown degrees of CS to confound the results of therapeutic trials in dogs. Behavioural responses have been assessed in order to evaluate potential alterations in nociceptive thresholds to heat, pressure, and cold (quantitative sensory testing, QST) in dogs experiencing OA and have indicated heat hypoalgesia [9], mechanical hyperalgesia [10,11] and cold hyperalgesia [11]. Limitations of QST methods in dogs have been reported as lack of feasibility in some dogs [9,12], effects due to the temperament and degree of anxiety of the dog [13], and the subjective interpretation of behavioural responses as an end point.

Nociceptive withdrawal reflexes (NWR) were first described as gross unified limb flexion movements in response to suprathreshold nociceptive stimulation by Sherrington [14]. Subsequent work however has characterised responses as co-ordinated activation and inhibition of both flexor and extensor muscles in a limb, with the resultant co-ordination dependent on the site of noxious stimulation [15,16]. NWR in individual hindlimb muscles can therefore be recorded using electromyography (EMG) in order to obtain a quantitative measurement of the motor response to a peripherally applied stimulus [17] and to study the organization of responses in individual muscles to stimulation at a particular site. Central sensitisation appears to enhance the protective movement provided by individual muscle NWRs [18]. Central sensitisation in chronic pain states in humans has been evaluated using NWR methodology [19], and CS has been demonstrated via NWR facilitation in experimental animal models of OA [20]. Measurement of NWR may be a more objective methodology than QST to gain insight into central nervous system plasticity in dogs and therefore may have utility in client-owned dogs with chronic pain conditions, including OA. Nociceptive withdrawal reflex methodology in client-owned dogs would also facilitate clinical trial evaluation of putative analgesics that target modulation of CS.

Previously published work has assessed NWR in conscious, trained research dogs, but client owned dogs are likely to be less tractable and artefacts associated with movement are likely to obscure EMG responses to nociceptive stimuli. Collection of EMG recordings of sufficient quality, whilst minimising the aversive experience associated with the stimulation (which, aside from animal welfare considerations, may, in itself, modulate nociceptive transmission [21,22]) may necessitate anaesthesia in such dogs. Recordings of EMGs elicited by both high frequency (200Hz) train-of-5 electrical stimuli [23], and lower frequency (2, 5 and 20 Hz) repeated stimuli [24] have been described in pain free conscious and acepromazine sedated [25] dogs; and proposed as an objective means of investigating central nervous system plasticity [25] as well as the effects of analgesic drugs [26]. Previous work has shown that a low dose (0.01mg kg^-1^) of acepromazine administered intravenously to experimental dogs produced mild tranquilisation for approximately 30 minutes and did not alter the characteristics of the recorded EMG [25]. However acepromazine sedation alone may not be considered to produce an appropriate degree of anxiolysis prior to the application of repeated nociceptive stimuli to client owned dogs. Sedation with dexmedetomidine may produce more effective anxiolysis but would be expected to affect NWR [27]. General anaesthesia would obviate any potential stress and awareness of nociception associated with NWR protocols, thus refining the procedure. It would also likely increase owner acceptability of the procedure, particularly if electrical stimuli are used. Preliminary pilot work investigating the ability to record EMGs in dogs anaesthetised with isoflurane, indicated that isoflurane concentrations required to prevent arousal abolished EMG responses in a significant number of tests. Alfaxalone anaesthesia has been employed in laboratory animal species to facilitate EMG recordings [20]. The specific aim of this current study was to develop a suitable alfaxalone based anaesthetic protocol for use in client owned dogs, which would enable us to record EMG data whilst minimizing movement artefact.

The primary outcome measures of this study were the magnitudes of the recorded EMGs; these were compared in order to determine the effects of sedation or anaesthesia produced by alfaxalone infusion on the recorded EMG, in comparison with results collected in acepromazine sedated dogs.

## Materials and Methods

Seven purpose bred male hound dogs (weight range 25.5–29.2 kg) were used for the NWR/EMG recordings. The dogs were housed individually within enclosures of dimensions approximately 1.5 x 4.6 metres, and 2.7 metres high, with enrichment consisting of toys that are changed out and rotated on a regular basis and standard bedding. The dogs’ exercise and socialisation program consisted of two 30 minute walks each day, outside, and two 20 minute play and socialization periods each day. The dogs were maintained on a standard complete dog food and food was withheld for eight hours prior to administration of sedative drugs. Ethical approval was obtained prior to beginning the study from the North Carolina State University Institutional Animal Care and Use Committee (IACUC; permit number 13-010-B). The experimental protocol was performed with the dogs restrained or anaesthetised in right lateral recumbency on a synthetic bedding, beneath which was a circulating warm water blanket and padded table top. Following completion of EMG recordings, carprofen (4mg kg^-1^) (Rimadyl, Zoetis, New Jersey, USA) was administered subcutaneously to treat any ongoing pain associated with the stimulation and recording protocols. Following completion of these experiments the dogs continued to be kept at the North Carolina State University Animal Care Facility to participate in further research projects.

### Placement of EMG recording electrodes

**Fig 1:**
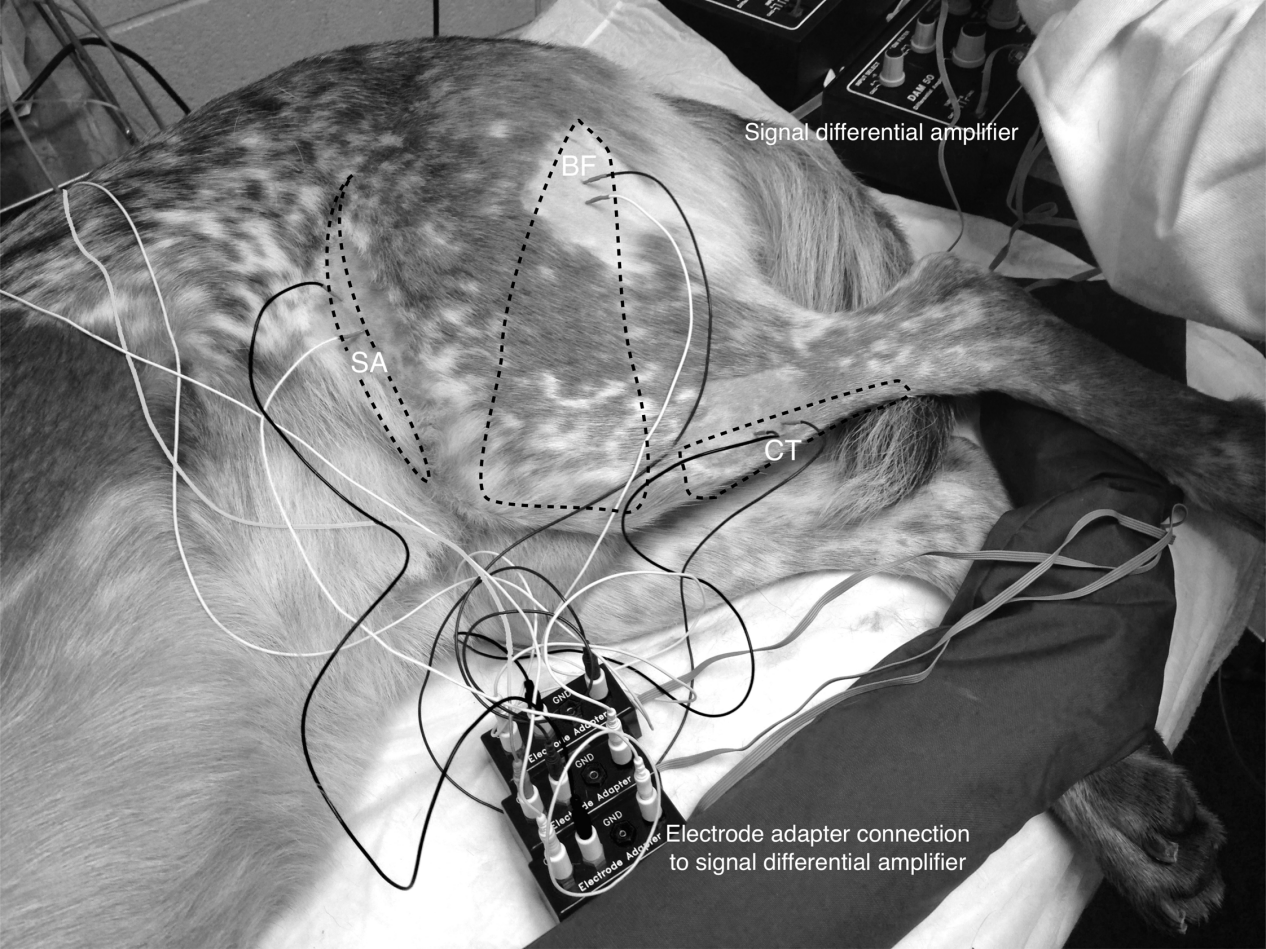
Demonstrating the position of recording electrodes in cranial tibial (CT), biceps femoris (BF) and sartorius (SA) muscles, together with ground electrode placement subcutaneously over the dorsal lumbar region.

**Fig 2:**
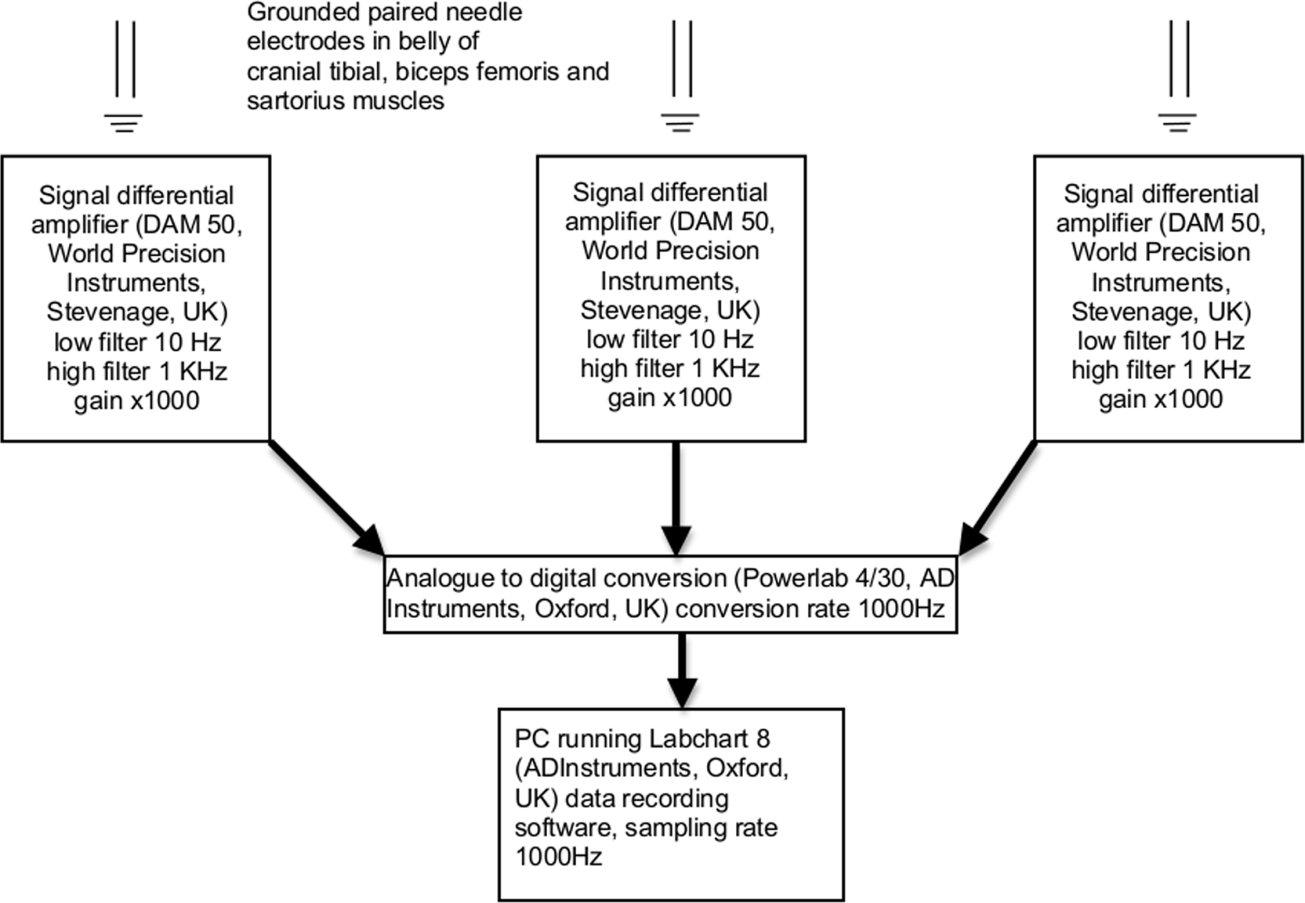
Schematic, illustrating recording connections.

The left pes was rested on a sandbag to support the limb perpendicular to the table top. Stainless steel needle recording electrodes (disposable subdermal needle electrode 12 x 0.40 mm, Natus Neurology Inc. Middleton, WI, USA) were placed transcutaneously, in pairs, into the belly of the cranial tibial (CT), biceps femoris (BF), and sartorius (SA) muscles of the left pelvic limb. A ground electrode for each pair of recording electrodes was inserted subcutaneously, dorsal to the dorsal spinous process of L6. The locations of the electrodes are shown in Fig 1. The distance between each of the electrodes in the pair was as follows: cranial tibial 18mm; biceps femoris 21mm; sartorius 21mm. Recording electrodes were connected to an analogue to digital converter (Powerlab 4/35, AD instruments, Oxford, UK) via separate differential amplifiers (DAM50, World Precision Instruments, Herts, UK) which applied a low pass filter of 10 Hz, high pass filter of 1 KHz, and gain of 1000 to the signal. The resulting digital signal was captured and recorded using Labchart 8 software (AD instruments, Oxford, UK) run on a Toshiba Tecra R850 (Toshiba Europe, 41460 Neuss, Germany) laptop, utilising Windows 7 operating system (Microsoft Corp., USA). Fig 2 is a schematic illustrating the recording connections used to measure NWRs.

### Sedation and anaesthesia

All dogs underwent the EMG recording procedure described below in each of three states: acepromazine sedation, alfaxalone sedation or alfaxalone anaesthesia.

In the acepromazine sedated state, dogs were administered acepromazine (Acepromazine Maleate Injection, Boehringer Ingelheim Vetmedica, Missouri, USA) 0.03 mg kg^-1^ injected intramuscularly into the lumbar longissimus dorsi, 30 minutes prior to being instrumented for EMG recording. The alfaxalone sedation state was produced by administration of an intravenous bolus of 1mg kg^-1^ alfaxalone (Alfaxan, Jurox, Missouri, USA), 30 minutes following acepromazine premedication as above. Sedation was maintained by intravenous infusion of alfaxalone (range 0.044–0.08 mg kg^-1^ min^-1^); rate of infusion was adjusted to produce a state in which dogs assumed lateral recumbency, were non-responsive to quiet auditory stimuli, and exhibited decreased muscle tone but maintained laryngeal reflexes. Dogs in this state were provided with supplemental oxygen at a rate of 1–2 litres min^-1^ via a close fitting facemask. The alfaxalone anaesthesia state was achieved by administration of an intravenous bolus of 1–2 mg kg^-1^ alfaxalone, 30 minutes following acepromazine premedication, as above, injected slowly until adequate conditions for orotracheal intubation were achieved. Anaesthesia was maintained by intravenous infusion of alfaxalone; rate of infusion was adjusted to produce a state in which dogs exhibited depressed muscle tone and laryngeal reflexes, sufficient to maintain orotracheal intubation with a cuffed endotracheal tube through which oxygen was delivered via a circle rebreathing system. The rate of alfaxalone infusion (range 0.075–0.1 mg kg^-1^ min^-1^) was adjusted to preserve a slow palpebral reflex. Following completion of EMG recordings, alfaxalone infusions were discontinued and dogs were monitored during recovery to the point that they were able to walk unaided, at which point they were transported in a wheeled crate back to their kennel. Examination the following day was performed to ensure no evidence of tissue damage or ongoing pain. Due to technical issues, dog 1 did not undergo testing in the alfaxalone sedation state and dog 5 did not undergo testing in the alfaxalone anaesthesia state.

### Stimulation protocol

#### Determination of the most sensitive area

**Fig 3:**
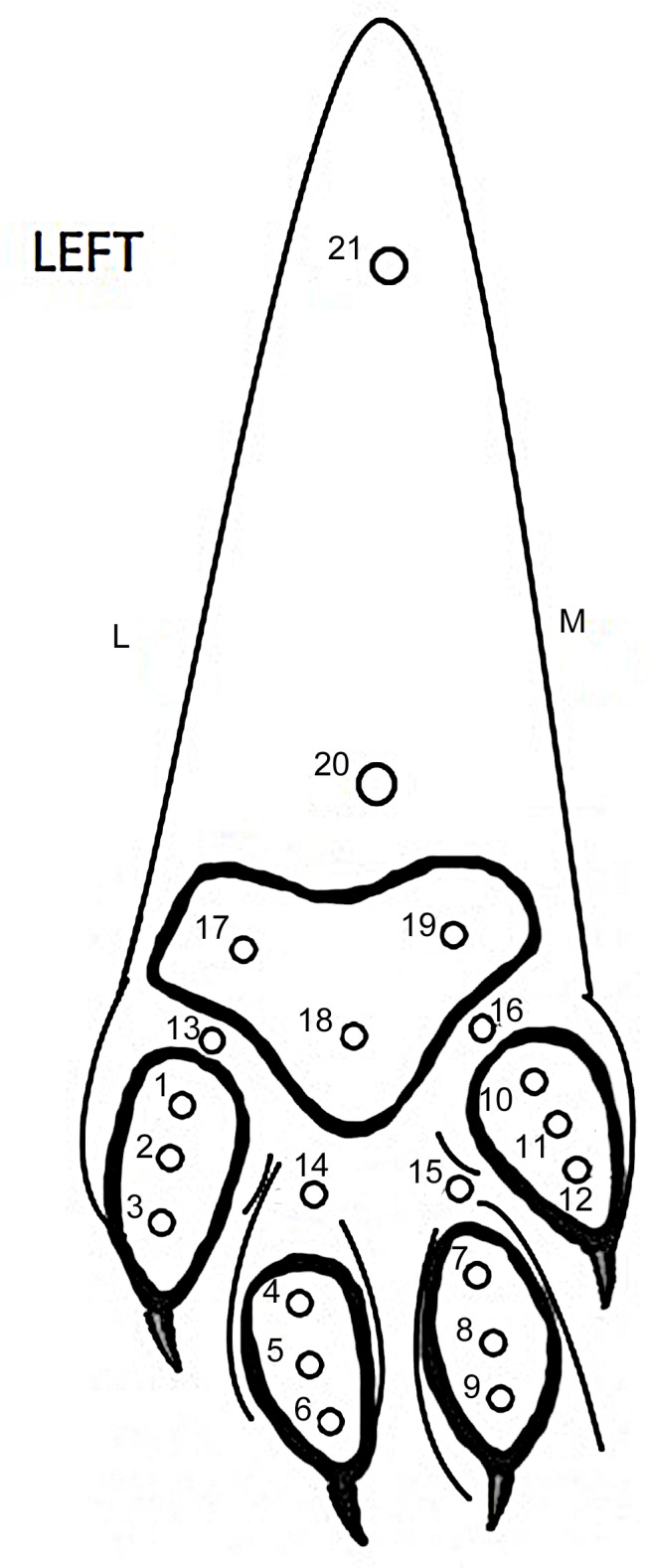
Diagram of left pes illustrating 21 points assessed for sensitivity by pinching with rat tooth forceps. L, lateral; M, medial.

Rat tooth forceps were used to deliver a firm pinch, sequentially to 21 points on the skin (Fig 3) on the plantar aspect of the left pes, whilst recordings of EMG activity were performed, to determine the most sensitive area (i.e. that which generated the largest magnitude EMG response) to a mechanical nociceptive stimulus. This sensitive area was then used to apply further mechanical and electrical stimuli. The results of this part of the experiment indicated that the most sensitive area was the area just proximal to the pad of the fourth digit (location 14 on Fig 3), and this area was used in subsequent stimulation protocols.

#### Mechanical Stimulus Response Curve

In order to construct an EMG stimulus response curve mechanical stimulation was delivered by the application of von Frey filaments (North Coast Medical, Inc., California, USA) in ascending weight order; 4, 6, 8, 10, 15, 26, 60, 100, 180 and 300g. Each filament was applied perpendicularly to the skin of the previously defined most sensitive area of the pes with a force just sufficient to cause the filament to bend. This position was maintained for 3 seconds, before removing the filament from contact with the skin. The complete series of von Frey filaments was applied in the same ascending order on a total of 3 occasions, with 5-minute intervals between each train of von Frey stimulation.

#### Response to Electrical Stimulation

Following completion of mechanical stimulation, a pair of stimulating needle electrodes (disposable subdermal needle electrode 12 x 0.40 mm, Natus Neurology Inc. Middleton, WI, USA) was placed into the plantar dermal tissues of the distal phalanx of the fourth digit, immediately proximal to the proximal edge of the digital pad, of the left pelvic limb. Electrical stimuli were delivered using a constant current stimulator from an isolated 100 V source (Stimulus isolator FE180, AD instruments, Oxford, UK).

(i) Electrical Stimulus Response Curve

One stimulus event comprised five 1 ms stimuli (Train-of-5, To5), which were delivered at a frequency of 100 Hz. In order to construct an EMG stimulus response curve, To5 events were triggered at 60-second intervals using currents of 0.1 (baseline), 1, 2, 3, 4, 5, 6, 7, 8, 9 and 10 mA. The complete series of stimulating currents were applied in the same ascending order on a total of 3 occasions, with 5-minute intervals between each occasion.

(ii) Response Stability Testing

For assessment of stability of the EMG response, 10 To5 events were delivered at 60- second intervals with a fixed current of 10 mA.

(iii) Temporal Summation

Subsequently, the effect of temporal summation was induced by a stimulus train, comprising eight single 10 mA stimuli delivered at a frequency of 1 Hz. This stimulation protocol was delivered on three occasions at five-minute intervals.

### EMG analysis

The mechanical and electrical stimulus intensity which elicited a greater than baseline amplitude response of CT, was noted and defined as nociceptive threshold. Post recording, 10Hz digital filtering was applied to the EMG traces, to further decrease movement artefact. EMG recordings were assessed visually to identify the time intervals which represented an early (indicative of an A- fibre mediated [23]) and late (consistent with a C- fibre mediated) response. The early period was defined as 0–100 ms following stimulation, while the late period was defined as 100–500 ms following stimulation. The integral of the rectified EMG response was extracted for each stimulus. Natural log(ln) [integral] values were subsequently analysed using the statistics package MLwiN [28] which allowed the repeated measures structure of the data to be properly accommodated within the analyses. The statistical model was based on a structure consisting of an individual measurement within repetition, within each anaesthetic state, within dog and then the effect of anaesthetic state and magnitude of stimulation (electrical or mechanical) was tested within a general linear model. To test for a non-linear relationship a series of polynomial terms for the stimulation were specified. Terms were retained within the models if they were significant at α ≤ 0.05 and were tested against a change in the log likelihood using a Chi square distribution. The resulting significant models were presented as graphs of the ln response plotted against the level of stimulation in order to depict the mean response to the stimulation.

The effect sizes and *P* values of the predictor variables included in the final models were tabulated, allowing reconstruction of the figures from the predictive equations.

Duration of testing procedure, and median nociceptive thresholds, were compared between the three states using Krukal-Wallis test, and Dunn’s multiple comparison test post-hoc.

## Results

The median (range) duration of each recording session for each state was 200 (165–270) minutes, and there were no differences in recording duration between the three states (*P* = 0.12).

During acepromazine sedation EMG activity was invariably associated with gross movement with flexion of the hock, stifle and hip. During alfaxalone sedation and anaesthesia EMG activity was associated with small contractions of the cranial tibial muscle (visible as ‘twitches’). EMG responses from the biceps femoris and sartorius muscles were infrequently recorded in all except the acepromazine sedated dogs. However it was possible to reliably and consistently record EMG responses from the cranial tibial muscle in each dog during each state of sedation or anaesthesia and so comparisons of data recorded were limited to the cranial tibial muscle.

### Tolerability of the protocol

Two dogs exhibited signs of anxiety (lip licking, ears drawn back, attempting to escape) during the stimulation protocol whilst sedated with acepromazine. Electrode displacement requiring electrode repositioning was encountered during electrical stimulation in these 2 dogs during the acepromazine sedated state, due to instances of gross movement, but in none of the dogs during alfaxalone sedation or anaesthesia. EMG recordings were feasible in the two anxious dogs following reassurance by handlers.

### Mechanical nociceptive threshold

Mechanical thresholds for individual dogs in each state, together with group medians, are shown in Table 1. Both alfaxalone treated groups exhibited significantly higher median mechanical thresholds (*P* = 0.01) compared to acepromazine sedated dogs, but thresholds were not different between alfaxalone groups.

**Table 1:** Mechanical and electrical threshold recordings from the three states. *P = 0.01 compared to acepromazine threshold.

State	Dog number	Mechanical threshold g	Median mechanical threshold g	Electrical threshold mA	Median electrical threshold mA
Acepromazine sedation	1	15	15	2.5	2
	2	60		2	
	3	8		1	
	4	26		2	
	5	100		6	
	6	15		1.5	
	7	8		2	
Alfaxalone sedation	2	300	240*	7	5.5*
	3	60		4	
	4	180		3	
	5	300		4.5	
	6	300		8	
	7	180		6.5	
Alfaxalone anaesthesia	1	300	300*	3	3.5
	2	300		10	
	3	300		7	
	4	180		2	
	6	60		4	
	7	300		2	

### Mechanical stimulation (von Frey) stimulus response curve

The effect sizes and *P* values of the predictor variables included in the final models are presented in S1 Table.

**Fig 4:**
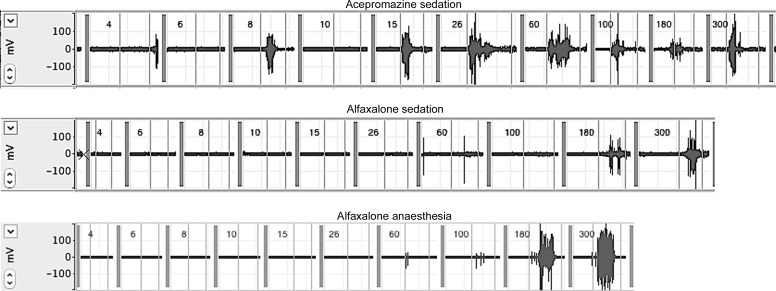
EMG recordings from cranial tibial muscle of dog 3 in response to application of increasing weights of von Frey filaments. Upper trace following acepromazine sedation, middle trace following alfaxalone sedation and lower trace following alfaxalone anaesthesia. Weights of von Frey filaments are shown on each trace in grams, thin grey vertical lines represent the period during which stimulation was applied at each weight.

**Fig 5:**
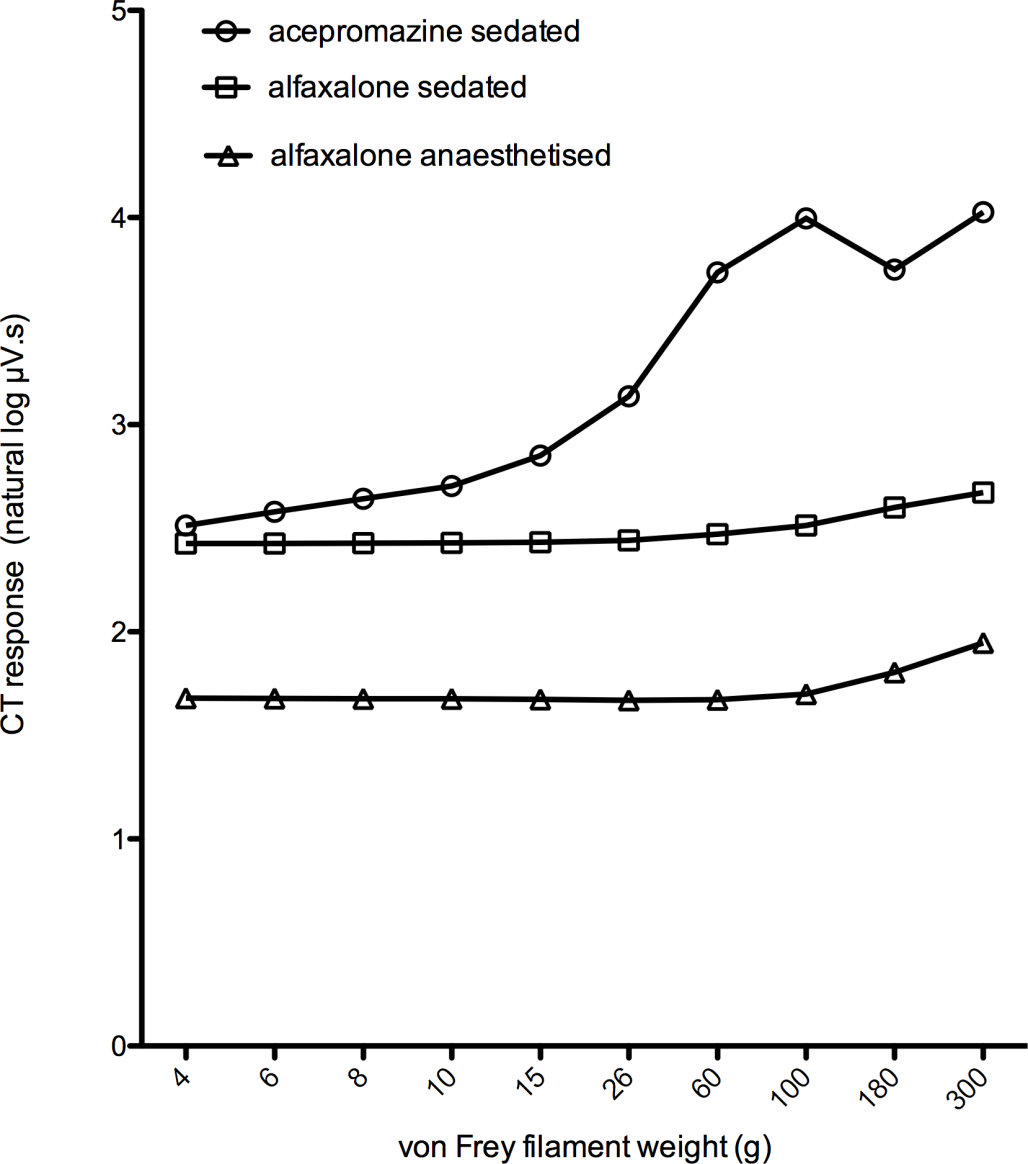
Responses to 3 second application of increasing weights of von Frey filament to the plantar skin of digit 4 in Fig 3.

Recorded responses increased in magnitude with increasing stimulus weight (S1 Table, Figs 4 and 5). Alfaxalone anaesthesia decreased the magnitudes of the EMG responses recorded (Fig 5), compared with those recorded in both acepromazine and alfaxalone sedated dogs, and there was a significant interaction between stimulus weight and state (*P* = <0.001, S1 Table). The repetition of the stimulus response curve was not identified as a significant source of response variability in the final model, indicating that neither sensitisation nor habituation to the stimulus occurred.

### Electrical nociceptive threshold

Electrical nociceptive thresholds for individual dogs in each state, and group medians, are shown in Table 1. Alfaxalone sedated dogs exhibited significantly higher median electrical thresholds (*P* = 0.01) compared to acepromazine sedated dogs, but thresholds were not different between alfaxalone groups, nor between acepromazine sedated and alfaxalone anaesthetised dogs. The characteristic response to electrical stimulation is illustrated in S1 Fig.

### Electrical stimulus response curves

**Fig 6:**
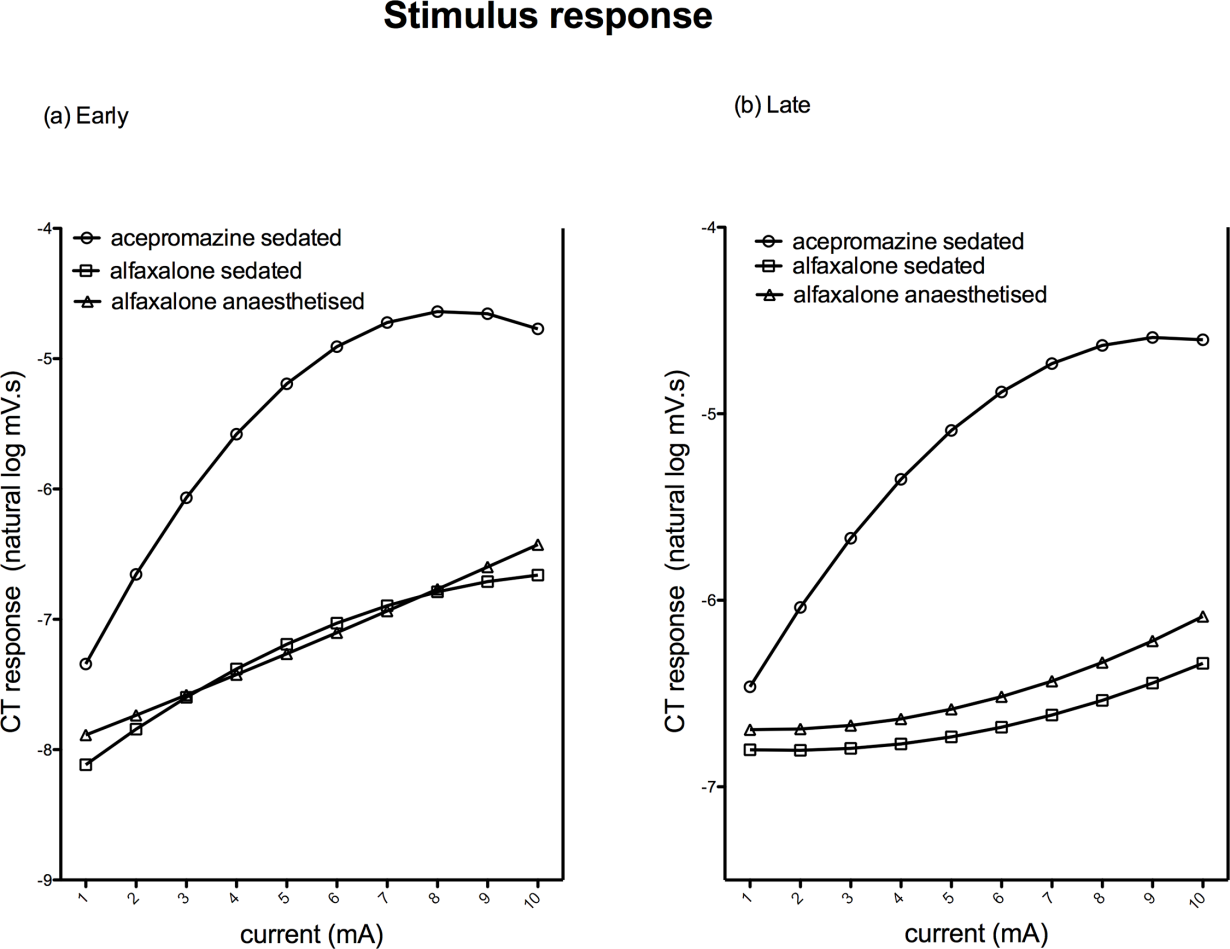
**Mean EMG responses to electrical stimulus response; early 0-100ms (a) and late 100-500ms (b).** Responses of the acepromazine sedated group are represented by ○, alfaxalone sedated by ◻, and alfaxalone anaesthetised by △.

Increasing magnitudes of responses to increasing stimulating current were observed in both early and late responses in all three states (S1 Table, Fig 6A and 6B).

There was a significant interaction between current and state (*P* = <0.001, S1 Table); whilst the rate of increase in EMG response with current was initially higher (greater increase in response per unit increase in stimulus intensity) in acepromazine sedated dogs compared to alfaxalone treated dogs, the magnitude of EMG responses began to plateau at currents greater than 6mA in acepromazine sedated dogs, but continued to increase up to the maximal (10mA) stimulation in both alfaxalone treated groups. Repetition and individual dog were not significant causes of variability in response.

### Electrical evoked response stability and comparison of EMG magnitude between states

**Fig 7:**
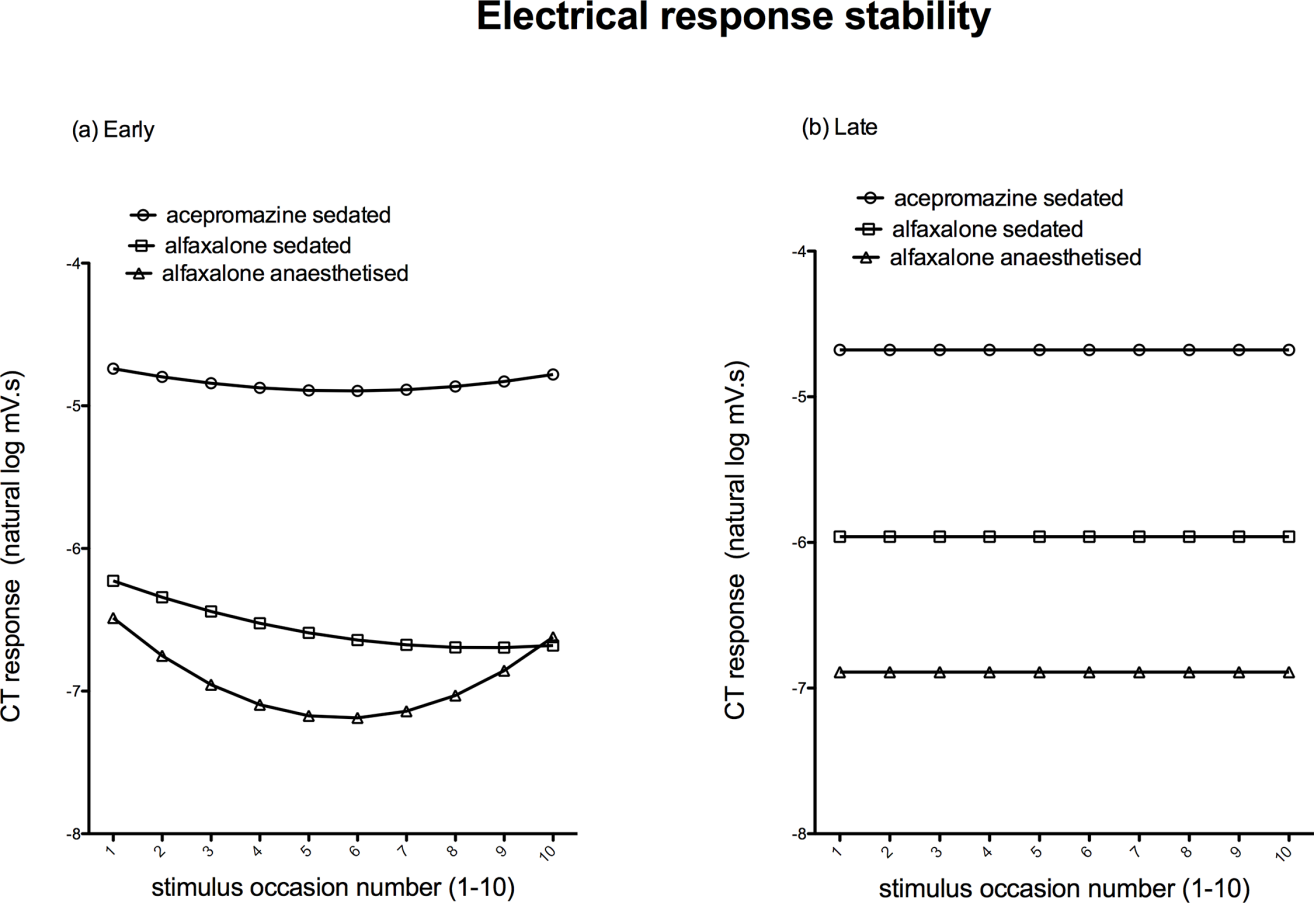
**Mean EMG responses to electrical stability; early 0-100ms (a) and late 100-500ms (b).** Responses of the acepromazine sedated group are represented by ○, alfaxalone sedated by ◻, and alfaxalone anaesthetised by △.

The early and late EMG responses were repeatable (there was no significant difference in EMG magnitude between subsequent stimulus occasions) in acepromazine and alfaxalone sedated dogs (Fig 7A and 7B). However there was significant variation of the magnitude of early (but not late) EMG response with occasion in alfaxalone anaesthetised dogs (*P* = 0.005, S1 Table).

Compared to acepromazine sedated dogs, alfaxalone sedation and anaesthesia significantly decreased the magnitude of early and late EMG (P = 0.007, S1 Table).

### Temporal Summation

**Fig 8:**
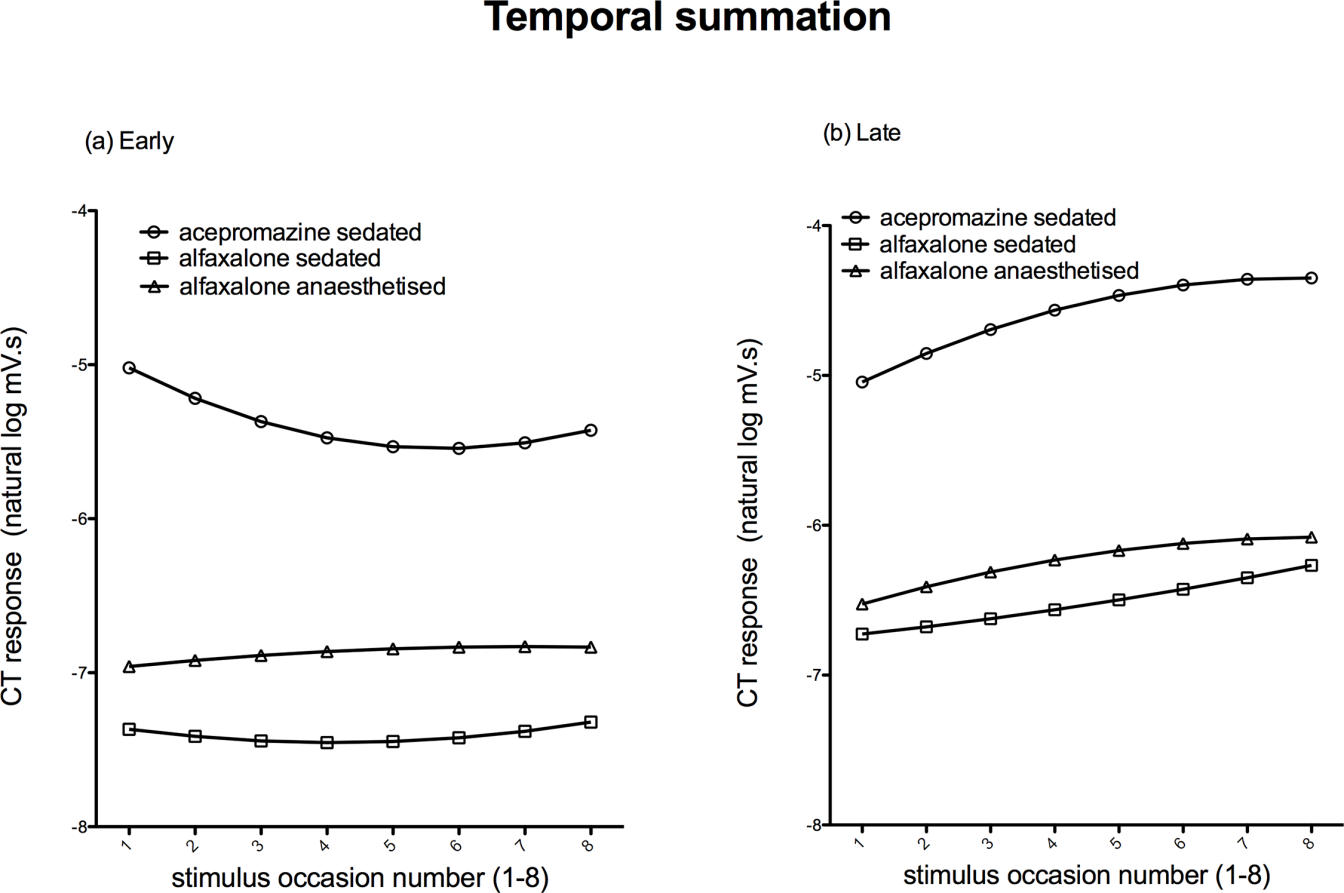
**Mean EMG responses to temporal summation; early 0-100ms (a) and late 100-500ms (b).** Responses of the acepromazine sedated group are represented by ○, alfaxalone sedated by ◻, and alfaxalone anaesthetised by △.

The magnitude of the early EMG response (Fig 8A) to the temporal summation stimulation protocol decreased with increasing occasion number in the acepromazine sedated dogs, yet remained constant with increasing occasion in both alfaxalone states. The magnitude of the late EMG response (Fig 8B) increased in all groups at a comparable rate (slopes are parallel). Compared to the acepromazine sedated group, the magnitude of the early and late EMG responses recorded in the alfaxalone sedated and anaesthetised groups was significantly decreased (*P* = <0.001, S1 Table).

There was an obvious difference in behavioural response of the dogs in the different states to temporal summation, with the acepromazine sedated dogs maintaining their stimulated limb in flexion with repeated delivery of the stimulus.

## Discussion

We have demonstrated that it is possible to record robust and repeatable EMGs in response to mechanical and electrical nociceptive stimuli in dogs administered alfaxalone. This represents the first investigation of the effect of anaesthetic state on recorded EMG responses in dogs and provides a methodological platform for future studies examining CS in dogs with naturally occurring OA pain. Alfaxalone anaesthesia decreased the magnitude of EMGs recorded in response to mechanical stimuli, and both alfaxalone treatments decreased the magnitude of EMGs in response to electrical stimuli, compared to EMGs recorded in dogs sedated with acepromazine only. However, responses remained readily identifiable, and continued to demonstrate expected stimulus response and temporal summation characteristics during both alfaxalone states. Furthermore there were no significant differences between the magnitudes of response to electrical stimulation between the two alfaxalone treatment groups. Thus, if NWR/EMG responses are to be recorded in client owned animals, general anaesthesia may be successfully employed in order to facilitate recording, and negate the aversive experiences associated with the stimuli. However, the stability data suggest that the magnitude of EMG response varies with anaesthetic state, therefore our results indicate that maintaining a stable plane of anaesthesia is critical for NWR/EMG recording, and is most likely to be achieved by continuous rate, or appropriately modelled target controlled, infusion. Generation and recording of EMG responses to electrical nociceptive stimuli in acepromazine sedated, pain free dogs replicates previous findings [25], supporting the contention that such a technique may be able to be transferred to the clinical setting to describe nociceptive processing in individual pet dogs. However behavioural signs, which could indicate anxiety, and electrode displacement resulting from subject movement, were noted more frequently in this state, which may limit the acceptability and utility of the procedure in client owned dogs. The recording protocol required the dogs to remain in lateral recumbency for 3 hours, which again may be more difficult to achieve without anaesthesia in client owned dogs, particularly those suffering from painful conditions. Whilst dog owners routinely consent to potentially aversive diagnostic testing in conscious dogs (e.g. venipuncture, fine needle aspiration of masses) these are expected to be brief experiences. A more prolonged procedure involving administration of repeated nociceptive stimuli may be expected to result in increased stress, compared to a momentary single aversive stimulus. Stress may inhibit [21] or potentiate [29] nociception. These psychological states represent potential confounding factors if NWR/EMG recording is attempted in non-anaesthetised dogs. There are a paucity of data available regarding the degrees of stress and welfare compromise involved in veterinary diagnostic procedures, however a semi-quantitative approach to assigning values to represent the welfare of dogs with respect to differing treatment options has been described [30]. A similar approach to assessing welfare impacts of differing diagnostic approaches could be employed, and in the absence of contra-indications, anaesthesia may represent a higher welfare method of achieving NWR evaluations.

Recording of EMG responses to nociceptive stimuli has previously been reported in dogs anaesthetised with isoflurane [27]; however our preliminary pilot work, using the methodology described above, indicated that isoflurane concentrations required to prevent arousal abolished EMG responses in a significant number of tests. Other investigators [31] reported that isoflurane anaesthesia depressed heat evoked withdrawal responses in rats, as measured by a force meter. In humans and ponies, NWR thresholds are significantly increased by isoflurane [32,33]. Isoflurane produces immobility at inspired concentrations of 0.8–1.2 minimum alveolar concentration, via preferential depression of ventral horn spinal neurones [34]. This property may limit its utility as an anaesthetic agent during procedures which require measurement of motor responses. Alfaxalone has previously been successfully employed as an anaesthetic agent during EMG recordings in rats [20,35] and, on this basis, was selected for this study in dogs. However the effects of this agent on NWR and EMG recordings have not been previously studied in dogs, and therefore both alfaxalone sedation and alfaxalone anaesthesia were studied.

Different modalities of nociceptive stimuli may have differing utility in the measurement of NWR/EMG recordings. Application of punctate mechanical stimuli to the area of the pes exhibiting highest sensitivity was performed in order to evaluate the responses to a physiologically relevant noxious stimulus which generates action potentials via activation of A- and C-fibre mechanically sensitive afferents. Both alfaxalone treatments significantly increased the threshold force required to elicit a response. In addition, the magnitude of response was decreased by alfaxalone anaesthesia, compared to acepromazine or alfaxalone sedation. To our knowledge this is the first study to determine EMG responses to punctate mechanical stimuli in dogs. Given that central sensitisation results in secondary hyperalgesia to mechanical, but not heat stimuli [36], assessment of EMG responses to mechanical stimulation may be used as a specific means of assessing and quantifying central sensitisation [20]. In order to evaluate mechanical sensitivity in anaesthetised dogs, stimuli of greater magnitude may be necessary. Application of electrical stimuli represents a quantifiable and widely employed (e.g. [25,37,38]), but non-physiological, means of eliciting NWRs. Electrical stimulation obviates the transduction required by physiological stimuli, and depolarises both low and high threshold nociceptive fibres simultaneously [39], in addition to non-nociceptive afferents [40,41]. Such widespread activation of different fibres may itself result in modulation of nociception at the spinal cord [40]. In acepromazine and alfaxalone sedated states, responses were repeatable, demonstrating that this protocol represents a stable condition, in which the effects of altering stimulus parameters can be assessed over the period of the experiment. Unexpectedly, the magnitude of the early response in the alfaxalone anaesthetised dogs varied with occasion (repeat of the To5). This is a concern, as such an effect may confound determination of NWRs. A 10m A stimulus was chosen to assess electrical stability on the basis that this would represent a suprathreshold stimulus. The response stability data for the alfaxalone anaesthetised state was derived from only 5 of the 7 dogs and the threshold current during alfaxalone anaesthesia in dog 2 was determined to be 10 mA. Re-examining the EMG recorded from dog 2 it is evident that a number of stimuli during the response stability testing had produced no response (i.e. 10 mA represented a subthreshold stimulus). Repeating the analysis, following exclusion of the stability data from dog 2 during the alfaxalone anaesthetised state, demonstrated that the responses of the remaining alfaxalone anaesthetised dogs were repeatable in this state, and suggests that the protocol should be equally valid in alfaxalone anaesthetised dogs.

Alfaxalone increased the threshold to electrical stimuli, although this was only statistically significant in the alfaxalone sedated state. Alfaxalone also decreased the magnitude of resulting EMGs. However the characteristics of increasing responses associated with increasing intensity of stimuli, allowing the construction of stimulus-response and temporal summation curves, were maintained. Decreased NWR threshold (i.e. a left shift in the stimulus response curve) has been reported in human populations experiencing augmented pain as a result of central sensitisation [19]; the ability to generate stimulus-response curves in pain-free alfaxalone infused dogs offers potential as a method of investigating central sensitisation in dogs. Further work to define reference EMG magnitude values to predetermined increasing electrical stimuli in normal, non-painful dogs would be beneficial. In all states, increasing late responses (consistent with C-fibre activation) were observed in response to temporal summation, suggesting this protocol would be suitable for investigating wind-up in alfaxalone infused dogs. The decreasing early EMG magnitude with increasing stimulus number in the acepromazine dogs is likely to reflect the behavioural response—continued maximal flexion of the limb—thus limiting the potential for further muscle contraction. Augmentation of NWR in response to temporal summation is regarded as a cardinal feature of central sensitisation, and has been reported in a number of clinical pain conditions in man [42,43].

Modular organisation of nociceptive withdrawal reflexes [16] produces greatest motor activity in specific muscles, activation of which would remove the stimulated area from the noxious stimulus. The most robust recordings in response to stimulation at point 14 were therefore made from the cranial tibial muscle, which would be the prime mover in flexing the tibiotarsal joint, removing the foot from the stimulus. It was not possible to record consistent EMG signals from the biceps femoris and sartorius muscles which are situated more distant to the site of stimulation. Higher intensity stimulation (e.g. temporal summation) did result in flexion of the hip and generation of EMG recordings from the sartorius (hip flexor) during acepromazine sedation, however responses were not observed in this muscle during alfaxalone infusions; comparisons were not, therefore, performed.

In conclusion, it was possible to record robust and reproducible EMG responses to electrical stimulation in both the alfaxalone states, and the results of the stimulus response and temporal summation stimulations were not different between the two states. Performing recordings in the alfaxalone anaesthesia state does not appear to negatively impact the data collected, and this state permits tracheal intubation, which is desirable in anaesthetised dogs. Following acepromazine premedication, induction of anaesthesia with 1–2 mg kg^-1^ alfaxalone, followed by a continuous rate infusion in the range 0.075–0.1 mg kg^-1^ min^-1^ has the potential to enable assessment of spinal nociceptive processing, by applying mechanical and electrical stimulus response, and electrical temporal summation protocols, in client owned dogs without subjecting them to potentially aversive experiences. Altered central nervous system nociceptive processing has been identified in a number of chronic pain conditions [44], and a means of investigating the nociceptive system may assist clinicians in targeting therapeutic interventions in individual animals.

## Supporting Information

S1 Fig Illustration of typical response evoked by train-of-five electrical stimulation and time domains during which measurements were performed. (TIF)

S1 Table Effect sizes and *P* values of the predictor variables (and interactions between variables) included in the final models. (DOCX)
