# Supplementary figures and images for: Alfaxalone Anaesthesia Facilitates Electrophysiological Recordings of Nociceptive Withdrawal Reflexes in Dogs (Canis familiaris)
Source: PLoS One. 2016 Jul 19;11(7):e0158990. doi: 10.1371/journal.pone.0158990 (PMC4951135; doi:10.1371/journal.pone.0158990)

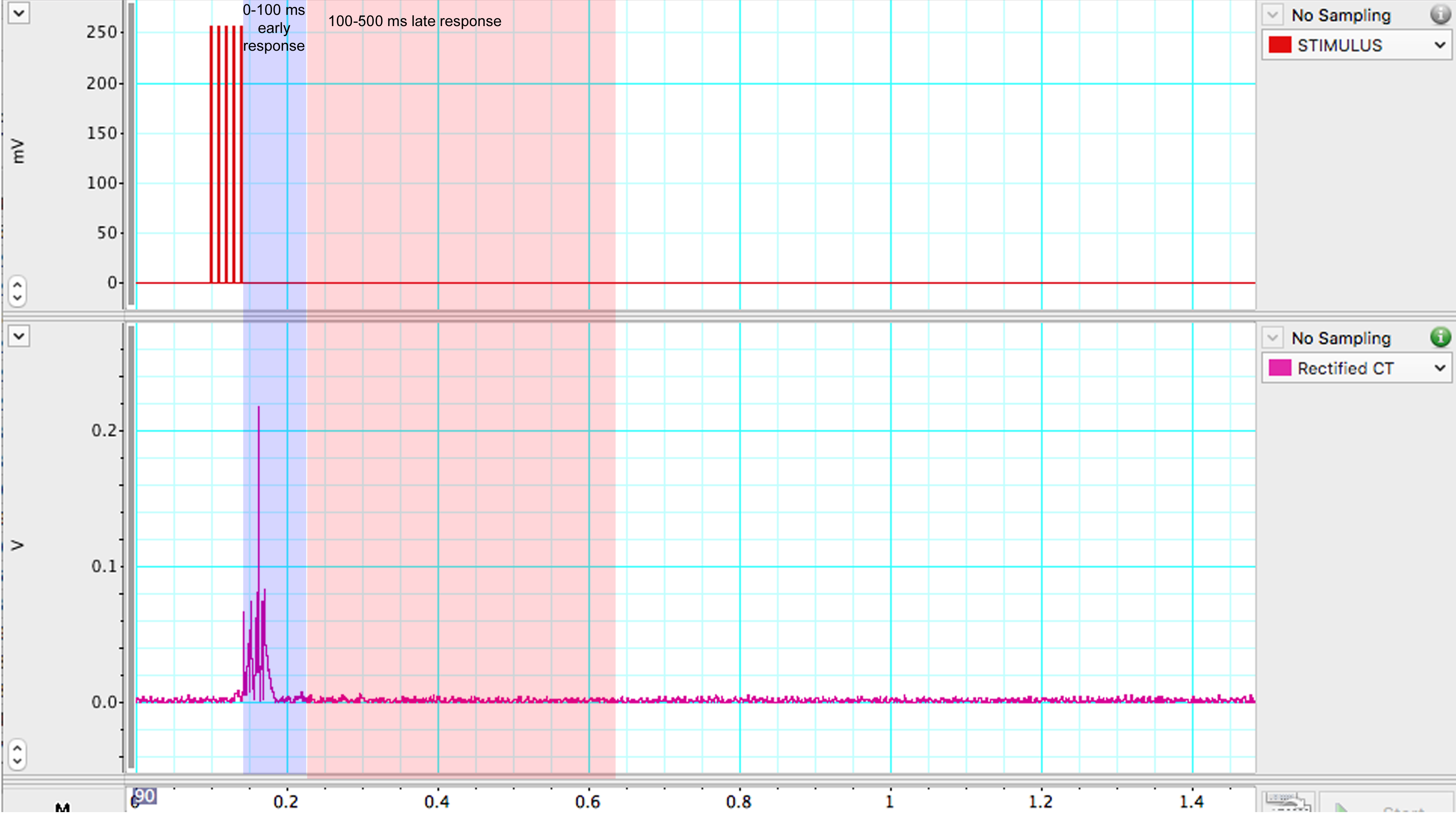

Supplement: S1 Fig — (TIF) [file pone.0158990.s001.tif]
