# Supplementary material for: Alfaxalone Anaesthesia Facilitates Electrophysiological Recordings of Nociceptive Withdrawal Reflexes in Dogs (Canis familiaris)
Source: PLoS One. 2016 Jul 19;11(7):e0158990. doi: 10.1371/journal.pone.0158990 (PMC4951135; doi:10.1371/journal.pone.0158990)
Supplement: S1 Table — (DOCX) [file pone.0158990.s002.docx]

Table S1 Effect sizes and *P* values of the predictor variables (and interactions between variables) included in the final models

| **Predictor variable** | **Mechanical** | |  | **Electrical stimulus (early response)** | | | **Electrical stimulus (late response)** | |  | **Electrical stability (early response)** | |  | | **Electrical stability (late response)** | | | **Temporal summation (early response)** | | | **Temporal summation**  **(late response)** | | |
| --- | --- | --- | --- | --- | --- | --- | --- | --- | --- | --- | --- | --- | --- | --- | --- | --- | --- | --- | --- | --- | --- | --- |
|  | **Ln(µV.S)** | **S.E.** | **P** | **Ln(mV.S)** | **S.E.** | **P** | **Ln(mV.S)** | **S.E.** | **P** | **Ln(mV.S)** | **S.E.** | | **P** | **Ln(mV.S)** | **S.E.** | **P** | **Ln(mV.S)** | **S.E.** | **P** | **Ln(mV.S)** | **S.E.** | **P** |
| **Constant, recorded during ACP sedation (x=0)** | 2.3773069 | 0.2941749 | - | -8.132 | 0.285 | - | -6.945 | 0.207 | - | -4.667 | 0.334 | - | | -4.678 | 0.346 | - | -4.775 | 0.313 | - | -5.264 | 0.287 | - |
| **Adjustment to constant by alfaxalone sedation** | 0.0461343 | 0.3698821 | 0.901 | -0.283 | 0.314 | 0.367 | 0.161 | 0.260 | 0.536 | -1.427 | 0.489 | 0.004* | | -1.281 | 0.474 | 0.007* | -2.528 | 0.441 | <0.001* | -1.504 | 0.442 | <0.001* |
| **Adjustment to constant by alfaxalone anaesthesia** | -0.6934438 | 0.3928979 | 0.078 | 0.095 | 0.314 | 0.762 | 0.264 | 0.260 | 0.310 | -1.491 | 0.512 | 0.004* | | -2.211 | 0.502 | <0.001* | -2.232 | 0.417 | <0.001* | -1.393 | 0.420 | <0.001* |
| **Mechanical stimulus weight** | 0.0349592 | 0.0035281 | <0.001* | - | - | - | - | - | - | - | - | - | | - | - | - | - | - | - | - | - | - |
| **Mechanical stimulus weight^2^** | -0.0002324 | 0.0000330 | <0.001* | - | - | - | - | - | - | - | - | - | | - | - | - | - | - | - | - | - | - |
| **Mechanical stimulus weight^3^** | 0.0000004 | 0.0000001 | <0.001* | - | - | - | - | - | - | - | - | - | | - | - | - | - | - | - | - | - | - |
| **Alfaxalone sedation x Stimulus weight** | -0.0343866 | 0.0051932 | <0.001* | - | - | - | - | - | - | - | - | - | | - | - | - | - | - | - | - | - | - |
| **Alfaxalone anaesthesia x Stimulus weight** | -0.0358420 | 0.0054657 | <0.001* | - | - | - | - | - | - | - | - | - | | - | - | - | - | - | - | - | - | - |
| **Alfaxalone sedation x Stimulus weight^2^** | 0.0002368 | 0.0000486 | <0.001* | - | - | - | - | - | - | - | - | - | | - | - | - | - | - | - | - | - | - |
| **Alfaxalone anaesthesia x Stimulus weight^2^** | 0.0002451 | 0.0000511 | <0.001* | - | - | - | - | - | - | - | - | - | | - | - | - | - | - | - | - | - | - |
| **Alfaxalone sedation x Stimulus weight^3^** | -0.0000005 | 0.0000001 | <0.001* | - | - | - | - | - | - | - | - | - | | - | - | - | - | - | - | - | - | - |
| **Alfaxalone anaesthesia x Stimulus weight^3^** | -0.0000005 | 0.0000001 | <0.001* | - | - | - | - | - | - | - | - | - | | - | - | - | - | - | - | - | - | - |
| **Stimulus occasion** | - | - | - | - | - | - | - | - | - | -0.078 | 0.064 | 0.223 | | - | - | - | -0.268 | 0.062 | <0.001* | 0.235 | 0.056 | <0.001* |
| **Stimulus occasion^2^** | - | - | - | - | - | - | - | - | - | 0.007 | 0.006 | 0.243 | | - | - | - | 0.023 | 0.007 | 0.001* | -0.015 | 0.006 | 0.012* |
| **Alfaxalone sedation x Occasion** | - | - | - | - | - | - | - | - | - | -0.062 | 0.096 | 0.518 | | - | - | - | 0.195 | 0.093 | 0.036* | -0.196 | 0.085 | 0.021* |
| **Alfaxalone anaesthesia x Occasion** | - | - | - | - | - | - | - | - | - | -0.281 | 0.099 | 0.005* | | - | - | - | 0.319 | 0.089 | <0.001* | -0.095 | 0.081 | 0.241 |
| **Alfaxalone sedation x Occasion^2^** | - | - | - | - | - | - | - | - | - | 0.001 | 0.008 | 0.901 | | - | - | - | -0.015 | 0.010 | 0.134 | 0.018 | 0.009 | 0.046* |
| **Alfaxalone anaesthesia x Occasion^2^** | - | - | - | - | - | - | - | - | - | 0.025 | 0.009 | 0.005* | | - | - | - | -0.027 | 0.010 | <0.001* | 0.007 | 0.009 | 0.437 |
| **Stimulus current** | - | - | - | 0.840 | 0.065 | <0.001* | 0.508 | 0.042 | <0.001* | - | - | - | | - | - | - | - | - | - | - | - | - |
| **Stimulus current^2^** | - | - | - | -0.050 | 0.006 | <0.001* | -0.027 | 0.004 | <0.001* | - | - | - | | - | - | - | - | - | - | - | - | - |
| **Alfaxalone sedation x Current** | - | - | - | -0.525 | 0.095 | <0.001* | -0.532 | 0.062 | <0.001* | - | - | - | | - | - | - | - | - | - | - | - | - |
| **Alfaxalone anaesthesia x Current** | - | - | - | -0.691 | 0.095 | <0.001* | -0.529 | 0.062 | <0.001* | - | - | - | | - | - | - | - | - | - | - | - | - |
| **Alfaxalone sedation x Current^2^** | - | - | - | 0.036 | 0.008 | <0.001* | 0.034 | 0.005 | <0.001* | - | - | - | | - | - | - | - | - | - | - | - | - |
| **Alfaxalone anaesthesia x Current^2^** | - | - | - | 0.052 | 0.008 | <0.001* | 0.035 | 0.005 | <0.001* | - | - | - | | - | - | - | - | - | - | - | - | - |
